# Eating disorders, primary care, and stigma: an analysis of research trends and patterns

**DOI:** 10.3389/fpsyt.2023.1243922

**Published:** 2023-09-29

**Authors:** Hatice Kurdak, Erkan Tiyekli, Sevgi Özcan, Zeliha Yelda Özer, Ayşe Nur Topuz

**Affiliations:** ^1^Department of Family Medicine, Faculty of Medicine, Çukurova University, Adana, Türkiye; ^2^Department of Information Technology, Çukurova University, Adana, Türkiye

**Keywords:** eating disorder, primary care, stigma, bibliometric analysis, topic analysis

## Abstract

Eating disorders (EDs) are a growing concern affecting millions worldwide. Early detection and treatment are crucial, but stigma can prevent people from seeking help. Primary care providers can play a critical role in early detection by coordinating care with other professionals. Understanding the research landscape on EDs, primary care, and stigma is essential for identifying knowledge gaps to direct future research and improve management. In this study, we aimed to analyze the scientific trends and patterns in research about EDs, primary care, and stigma. A bibliometric analysis was conducted using the Web of Science database to collect articles published between May 1986 and May 2023. Bibliometric indicators were utilized to examine authorship, collaboration patterns, and influential papers. Topic analysis was performed to identify stigma-related terms within the dataset. A total of 541 research articles were analyzed, and it was found that the average number of publications per year has increased linearly from nearly zero in 1986 to 41 in 2022. One of the study’s main findings is that despite this linear increase over the years, the subject of stigma did not take a prominent place in the literature. Only a few stigma concepts could be identified with the topic analysis. The authors in the field are also interested in; screening, neurotic symptoms, training, adolescent, obesity-related conditions, and family. One-third of all publications were from 15 journals. However, only two of them were primary healthcare journals. Leading authors’ collaborations were another critical finding from the network analysis. This may help to expand primary care related EDs research to end the mental health stigma. This study provides insights into the research trends and patterns regarding eating disorders, primary care, and stigma. Our findings highlight the need to address primary care’s impact and stigma on EDs. The identified research gaps can guide future studies to improve the prevention, diagnosis, and treatment of eating disorders in primary care settings.

## 1. Introduction

Eating Disorders (EDs) have gained increasing prevalence over time and are categorized into seven main groups in the latest edition of the Diagnostic and Statistical Manual of Mental Disorders (1). The Global Burden of Disease Study in 2019 reported that approximately 14 million people, including 3 million children and adolescents, were affected by ED (2). Early detection and treatment of EDs is crucial for symptom reduction, recovery, and the improvement of the overall prognosis. Primary care professionals may be pivotal in preventing, early diagnosing, and treating ED. However, studies have shown that appropriate treatment is often delayed, sometimes by months or even years, after the onset of symptoms. The delay in diagnosis and treatment of EDs has been recognized as a significant concern that needs to be addressed (3–6). Furthermore, EDs pose unique challenges as they can lead to serious health complications and are often accompanied by discrimination and stigma, which may prevent individuals from seeking help and exacerbate their condition. Stigma is a significant barrier for individuals face with mental health disorders; EDs are among the most stigmatized (7). People with EDs experience negative attitudes and prejudices in various aspects of their lives, hindering their ability to seek help and potentially leading to detrimental consequences, including suicide (8, 9).

In this context, healthcare providers should be aware of the stigma associated with EDs and be equipped to provide counseling and support in addressing this issue. Understanding the research landscape concerning ED, primary care, and stigma is crucial for identifying gaps in knowledge, promoting collaboration, and directing future research efforts (10).

Bibliometric analysis, a quantitative statistical approach to analyzing the literature, has become a valuable tool in examining the advancement of scientific knowledge and identifying research trends (11). By analyzing scientific publications on ED, primary care, and stigma, we can gain insights into the current state of research, the collaboration patterns among researchers, and potential future directions.

While several bibliometric studies have been conducted on ED, none have specifically focused on the primary care perspective and stigma (12–19). Therefore, this study aims to analyze the scientific trends and research patterns regarding ED, primary care, and stigma. The research questions of this study are as follows: (1) What are the general characteristics of the scientific trends on research related to primary care, eating disorders, and stigma? (2) What are the article characteristics and most productive authors in terms of countries, metrics, and citations in the field? (3) What are the social structure, and collaboration networks between authors and countries? (4) What are the implications and trends of the most frequent keywords, and the identified topics in the context of eating disorders (ED), and how have these topics evolved over time? By exploring these aspects, this study seeks to contribute to the understanding and advancing knowledge on ED, primary care, and stigma, ultimately aiming to improve the prevention, diagnosis, and treatment of these conditions.

## 2. Materials and methods

### 2.1. Data source and search strategy

The Web of Science (WoS) database is known as one of the most comprehensive databases worldwide, indexing high-quality journals widely used for scientometric analysis of scientific literature. Therefore, the WoS Core Collection (WoSCC) database was specifically used for this study. Based on our search using eating disorders and primary care-related keywords, it was found that the earliest article on the WoS was published in 1986. Therefore, the bibliometric analysis was conducted to collect articles published between May 1986 and May 2023.

### 2.2. Exclusion criteria

In order to analyze only original research articles, this study has excluded document types such as reviews, letters, meeting abstracts, and editorial materials. Additionally, articles that were published in languages other than English were also excluded.

### 2.3. Search keywords

Title, abstract, and keyword searches were performed using the following search terms:

#1. (“eating disorder*” OR “anorexia nervosa” OR bulimi* OR “binge eating” OR binge-eating OR “purging disorder” OR “night eating syndrome” OR “food intake disorder*” OR orthorexia OR pica OR “rumination disorder*”) AND
#2. (“primary care” OR “family medicine” OR “general practice”) AND
#3. (stigma* OR bias OR discriminat* OR prejudice OR stereotyp* OR victim* OR blame OR shame OR tease OR bully OR belief* OR assumption* OR attribution* OR attitude* OR perception* OR “body image” OR “self-esteem” OR “negative attitude*”).

The initial search yielded 208 documents. After applying the inclusion criteria, 167 articles were retained. Since the number is quite scarce for bibliometric analysis, a second round of search was conducted to expand the dataset by using only the search terms from #1 AND #2, resulting in 772 documents. Following the exclusion of reviews, letters, meeting abstracts, and editorial materials, 600 articles were reached. Finally, 32 non-English articles were excluded, leaving 568 articles for data analysis. In the WoS database, information such as article title, abstract, keywords, source, citation count, cited references, WoS categories, research areas, publisher information, and authors’ details (names, affiliations) were retrieved. The search was completed on June 4, 2023.

### 2.4. Bibliometric analysis

Bibliometric analysis is a quantitative method used to evaluate and measure various aspects of scientific literature. It involves the application of statistical and mathematical techniques to bibliographic data, such as publication records, citations, authorship, and keywords, to gain insights into patterns, trends, and relationships within a specific field of study. This analysis can provide valuable information about the impact, visibility, and collaboration patterns of researchers, institutions, and countries, as well as identify influential articles and key research topics. It is widely used in academic research to assess the scientific output, evaluate research performance, and identify emerging areas of interest. We used bibliometric indicators including citation counts, h-index, g-index, m-index, journal impact factor, co-authorship networks, and keyword co-occurrence analysis. These indicators can help to understand the influence and visibility of articles, the collaborative relationships among authors, the most influential journals, and the main themes and trends within a research field (20). The R-Studio (Version 4.3.0, released on April 21, 2023) was utilized for bibliometric and topic analysis of the scientific information related to EDs and primary care (21, 22). The analysis included the examination of authorship, citation counts, journals, countries, institutions, keywords, and summary statistics.

Utilizing Biblioshiny, a powerful tool designed in the R-Studio’s Bibliometrix package for processing CSV files, we analyzed the most frequently cited documents found within WoS, a comprehensive database (22). Examining the database’s citations allowed us to construct a network analysis among the various authors present. We used the Louvain algorithm to identify closely working groups of individuals to understand the collaboration between these authors better (23). This process requires basic network parameters as input, operates without restrictions, and extracts clusters automatically. In this analysis, the network parameters were established to be a maximum of 100 nodes, with at least two edges per node, and eliminating isolated nodes. In addition, centrality measures such as betweenness, closeness, and PageRank were employed to determine the significance of each author within the network. Although PageRank was initially developed for website ranking, it has also been used to rank authors effectively in recent years (24, 25).

### 2.5. Topic analysis

Topic modeling is a statistical model used to discover abstract topics in a collection of documents. It leverages machine learning and natural language processing techniques to uncover hidden semantic structures in text data. To identify stigma-related research topics within the dataset, topic modeling was performed using the title, abstract, and keywords. For topic analysis of text data, the texts are appropriately cleaned and standardized using the R programming language and the “tm” and “stringr” packages (26, 27). First, unnecessary characters, numbers, and punctuation marks in the text data were cleaned, uppercase letters were converted to lowercase letters, and stop words (e.g., “the,” “a,” “in,” “of,” “and,” “but”) were removed. The stemming process was applied by finding the roots of the words. In the abstract text, words such as “background” and “aim” were removed to create a more concise text. Following terms in the text were replaced by “stigma_related_words”: prejudices, weightbias, biases, stereotype, stereotypes, stereotypical, stereotypy, victim, victimization, victims, blame, shame, bullied, bullying, belief, beliefs, believe, believed, behaviors beliefs, assuming, assumption, assumptions, attributable, attributed, attribution, attributions, attitude, attitudes, perception, perceptions, selfesteem, self-image, esteem, self-acceptance, self-image, attitude, attitudes, attitudinal, meta-cognitive beliefs, inclusion beliefs, causal attribution, low self-esteem, negative affect, ebullying, evictimization, stigmatizing experiences, stigma, weight bias, shame, and beliefs. This modification aimed to enhance the focus on topics related to stigma and its associated factor. Finally, the process involved converting multi-word expressions into a single word. For example, “body mass index” was changed to “body_mass_index.”

Topical analysis was carried out on the cleared text data obtained with the Latent Dirichlet Allocation (LDA) model. The LDA model is a basic learning method used to identify different topics representing a combination of words frequently occurring in the text. The “lda” package of the R program was used for this purpose (28). The log-likelihood method evaluates the model’s fit to the data by calculating the log-likelihood values of the LDA model for different topic numbers (29, 30).

The log-likehood values extracted from the LDA calculations were computed using different number of topics included in the model. The results were used to find the optimum number of topics to be used. The created LDA model was used to determine the most frequent words for each topic and the weights of these words.

The syntaxes that are used to perform the bibliometric and topical data analysis and to generate the graphs are provided as Supplementary material 1, 2.

### 2.6. Ethical considerations

Ethical approval was not required for this study as the data used were obtained from a public database and did not involve human participants.

## 3. Results

### 3.1. General characteristics

**Figure 1:**
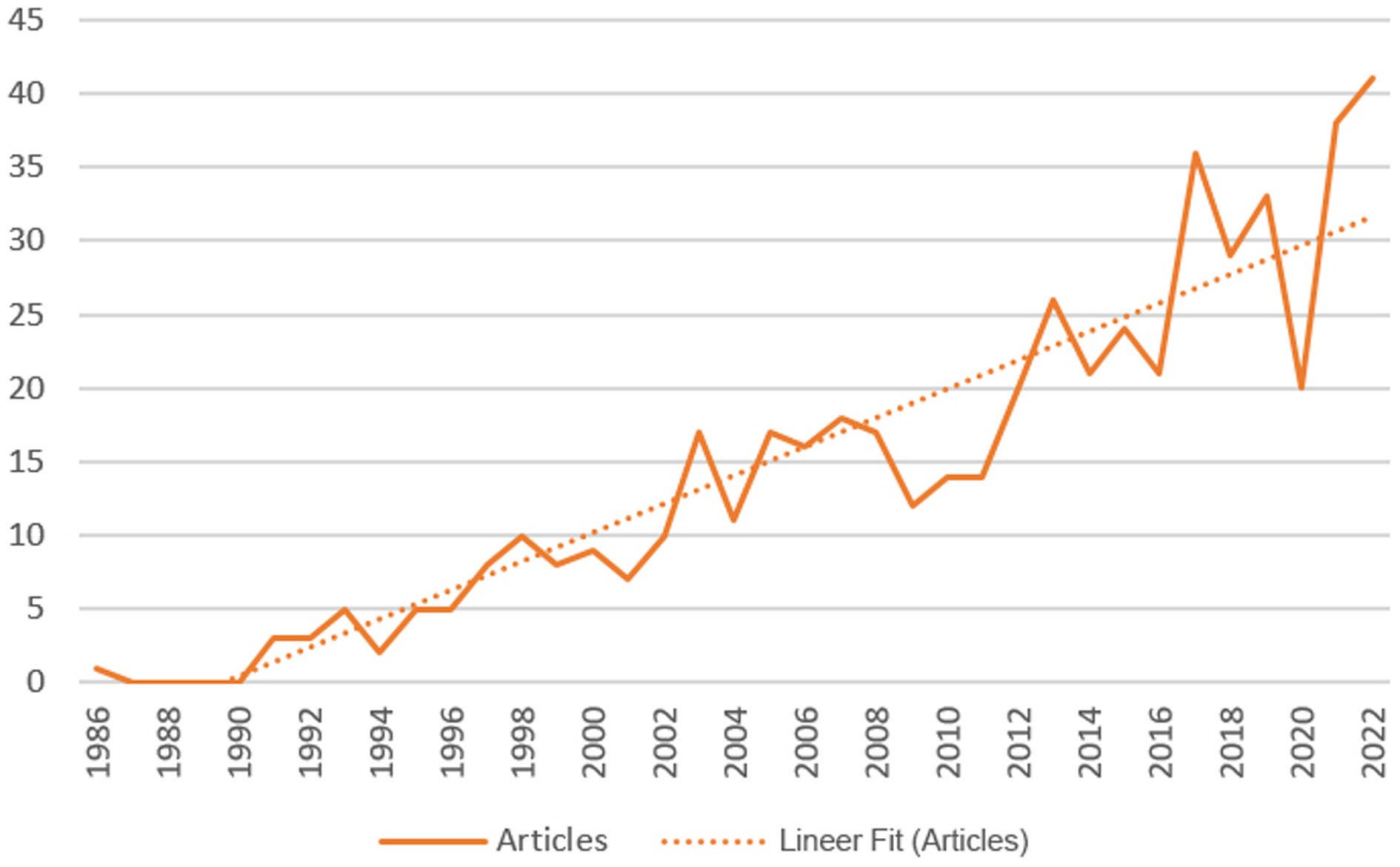
Annual scientific production of publications related to Primary Care and Eating Disorders from 1986 to 2022.

**Figure 2:**
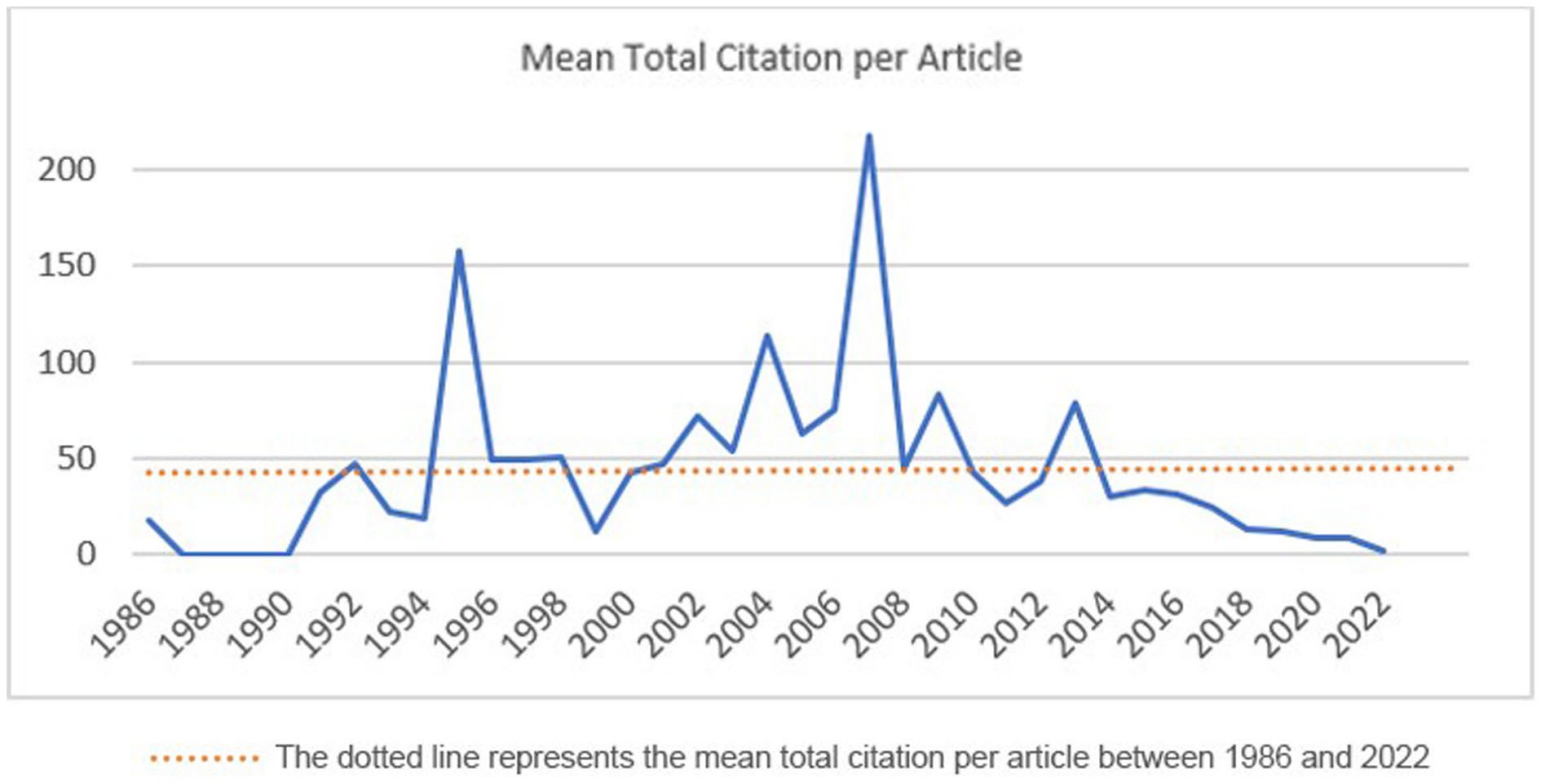
Annual citation trend of Primary Care Related Eating Disorder Publications from 1986 to 2022.

Over the years included in the scope of this study, 541 research articles were identified from different disciplines and fields. The oldest study was from 1986 and the latest one was in 2023. Five of the articles were early access. Overall, the number of authors who had contributed to the field was 2,062, the number of authors of single-authored documents was 34, co-authorship per document was 4.6, the total number of author’s keywords was 1,339, the total number of the references were 16,945, and the average citations per document were 40.84. The international co-authorships rate was 17.38% and the annual scientific original research publication growth rate was 8.43%. The mean number of articles published in the journals each year between 1986 and 2020 was 14.08 ± 11.31 (2023 was excluded from the average calculation as this year was not complete at the time of the data analysis). While the average annual number of publications prior to 2005 was less than 20, the number of publications increased to 41 in 2022 (Figure 1). The mean total citation per article peaked in 1995 and 2005 but declined dramatically under the average value after 2014 (Figure 2).

According to the Core Sources by Bradford’s Law analysis, one third of the publications were published in 15 journals and these journals made the most significant scientific contribution to the field. On the other hand, the second third of the publications were published in 62 journals and the last third were published in 180 journals (Table 1). The International Journal of Eating Disorders is the leading journal that publishes articles about primary care related EDs. Four of the journals among the top 25 are primary health care journals refined by the WoS Categories (American Family Physician, British Journal of General Practice, Primary Care, and Family Practice). Furthermore, according to the WoS Core Collection categories, the three most prominent publication categories were Psychiatry (43.44%), Psychology Clinical (28.84%), and Medicine General Internal (17.56%), while Primary Health Care (7.39%) ranked seventh (Figure 3).

**Table 1:** Top 25 most productive and influential sources listed by using Core Sources by Bradford’s Law analysis.

#	Sources	Number of articles	h_index	g_index	m_index	Total citations	Publication year-start	Zone
1	International Journal of Eating Disorders	57	23	39	0.852	1,640	1997	1
2	Journal of Eating Disorders	14	5	7	0.714	66	2017	
3	Eating Behaviors	13	9	13	0.600	218	2009	
4	European Eating Disorders Review	13	8	13	0.276	180	1995	
5	BMC Psychiatry	10	8	10	0.533	333	2009	
6	Pediatrics	10	9	10	0.321	900	1996	
7	Behavior Research and Therapy	9	9	9	0.450	1,331	2004	
8	American Family Physician	8	7	8	0.269	190	1998	
9	BMJ Open	8	5	8	0.455	336	2013	
10	Postgraduate Medicine	8	5	7	0.152	59	1991	
11	Psychological Medicine	8	8	8	0.242	569	1991	
12	Eating and Weight Disorders—Studies on Anorexia Bulimia and Obesity	7	3	4	0.300	22	2014	
13	British Journal of General Practice	6	6	6	0.188	214	1992	
14	British Journal of Psychiatry	6	6	6	0.194	538	1993	
15	Comprehensive Psychiatry	6	6	6	0.353	514	2007	
16	Early Intervention in Psychiatry	6	3	6	0.176	45	2007	2
17	Eating Disorders	6	4	6	0.333	71	2012	
18	General Hospital Psychiatry	6	6	6	0.545	126	2013	
19	Journal of Clinical Psychology in Medical Settings	6	5	6	0.238	107	2003	
20	Primary Care	6	4	6	0.154	50	1998	
21	Journal of Adolescent Health	5	4	5	0.160	131	1999	
22	Journal of Affective Disorders	5	4	5	0.211	305	2005	
23	Journal of Consulting and Clinical Psychology	5	5	5	0.357	154	2010	
24	Family Practice	4	3	4	0.176	53	2007	
25	Frontiers in Psychology	4	3	4	0.375	123	2016	

**Figure 3:**
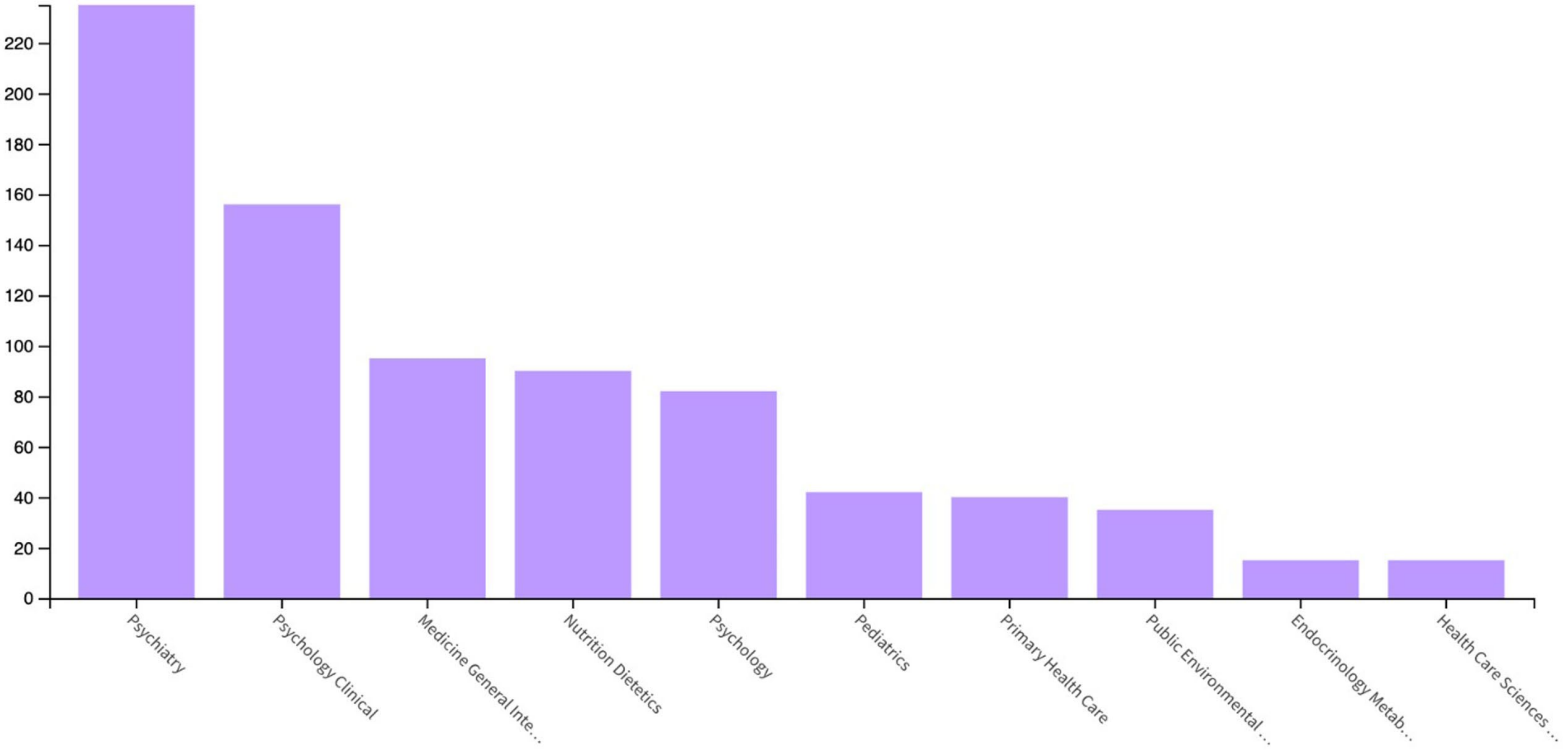
Top 10 publication categories according of papers related to Primary Care and Eating Disorders according to the Web of Science.

### 3.2. Article characteristics and most productive authors

**Figure 4:**
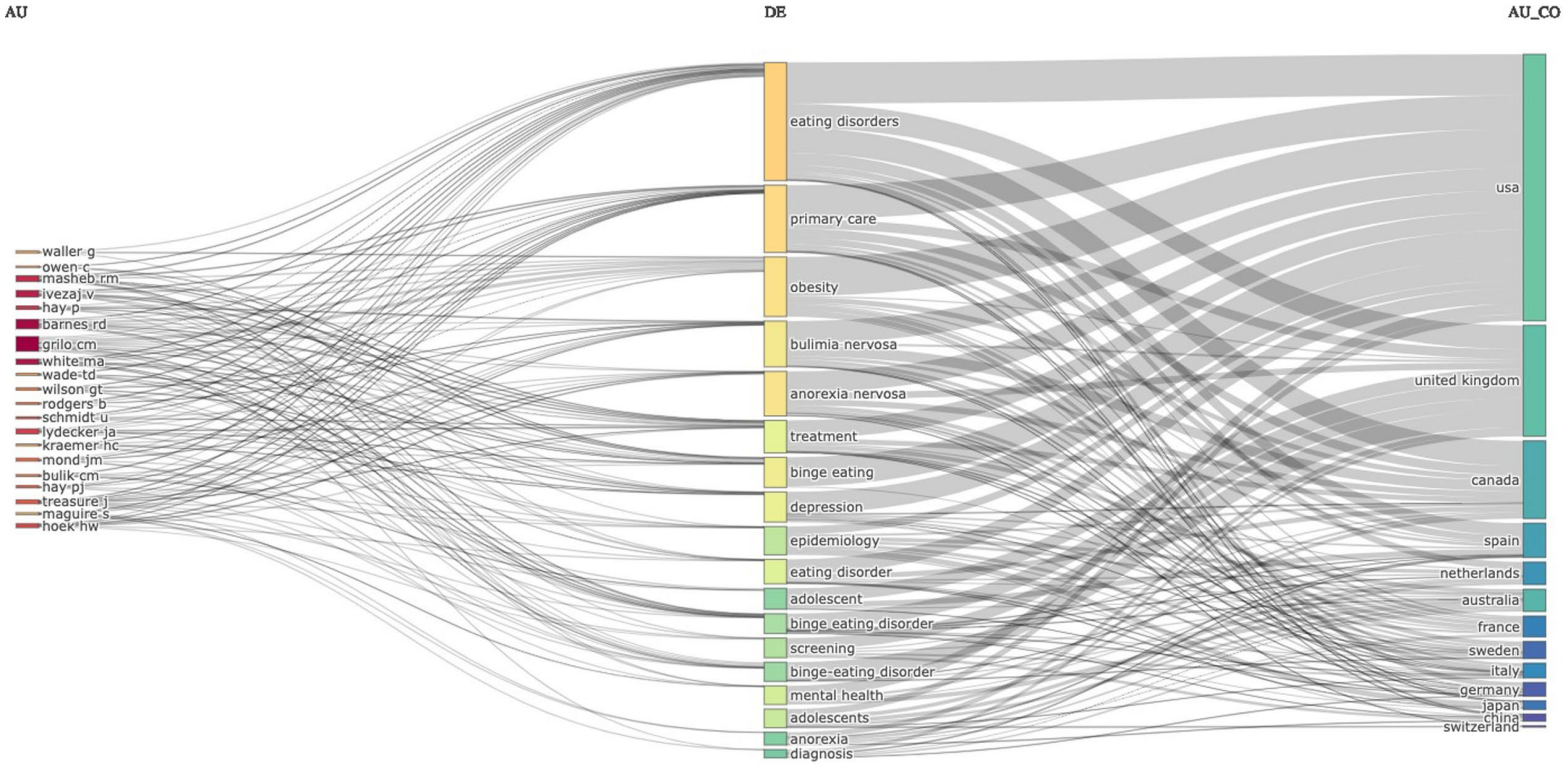
A Sankey diagram (three-field plot) visualization of the top countries leading research in the field by authors, the main research topics (keywords).

A list of the top 20 most cited documents according to local/global citation ratio and the top 10 most productive authors with high metrics and citations in the field are shown in Tables 2, 3 respectively. A Sankey diagram (Three-Field Plot) is used for the visualization of the top countries leading research in the field as well as the top authors and the main research topics (keywords). Thicker rectangles indicate greater frequency in the diagram. The thickness and the number of connecting nodes, inflows, and outflows indicate a larger number of connections (Figure 4).

**Table 2:** Top 20 most cited documents according to local and global citations (Ranked according to the local citations).

First Author	Title	Year	Citations		LC/GC^*^ (%)
			Local	Global	
Johnson JG	Health problems, impairment and illnesses associated with bulimia nervosa and binge eating disorder among primary care and obstetric gynecology patients	2001	65	177	36.72
Cotton MA	Four simple questions can help screen for eating disorders	2003	40	138	28.99
Mond JM	Screening for eating disorders in primary care: EDE-Q vs. SCOFF	2008	38	165	23.03
Mond JM	Validity of the Eating Disorder Examination Questionnaire (EDE-Q) in screening for eating disorders in community samples	2004	26	778	3.34
Walsh BT	Treatment of bulimia nervosa in a primary care setting	2004	20	85	23.53
Banasia K SJ	Guided self-help for bulimia nervosa in primary care: a randomized controlled trial	2005	18	59	30.51
Grilo CM	A randomized controlled comparison of guided self-help cognitive behavioral therapy and behavioral weight loss for binge eating disorder	2005	17	134	12.69
Hoek HW	Incidence, prevalence, and mortality of anorexia nervosa and other eating disorders	2006	17	520	3.27
Grilo CM	Treatment of binge eating disorder in racially and ethnically diverse obese patients in primary care: randomized placebo-controlled clinical trial of self-help and medication	2014	15	47	31.91
Hoek HW	The incidence and prevalence of anorexia nervosa and bulimia nervosa in primary care	1991	14	91	15.38
Hoek HW	Impact of urbanization on detection rates of eating disorders	1995	14	94	14.89
Parker SC	Eating disorders in graduate students: exploring the SCOFF questionnaire as a simple screening tool	2005	14	59	23.73
Striegel-Moore RH	Health services use in eating disorders	2008	14	84	16.67
Wilson GT	Cognitive–behavioral guided self-help for eating disorders: effectiveness and scalability	2012	14	120	11.67
Linville D	Medical providers’ self-perceived knowledge and skills for working with eating disorders: a national survey	2012	12	31	38.71
Grilo CM	Self-help for binge eating disorder in primary care: a randomized controlled trial with ethnically and racially diverse obese patients	2013	12	33	36.36
Freund KM	Detection of bulimia in a primary care setting	1993	9	25	36.00
Field AE	Prospective association of common eating disorders and adverse outcomes	2012	9	125	7.20
Grilo CM	Psychiatric disorder co-morbidity and correlates in an ethnically diverse sample of obese patients with binge eating disorder in primary care settings	2013	9	51	17.65
Barnes RD	A randomized controlled trial comparing scalable weight loss treatments in primary care	2014	9	31	29.03

^*^LC/GC: Local citations/Global citation.

**Table 3:** Top 10 most productive authors with high metrics with citations in the field.

Authors	Articles	Articles fractionalized	Local/Total citations	h_index	g_index	m_index	Publication year start
Grilo CM	29	8.74	77/739	17	27	0.895	2005
Barnes RD	19	4.99	46/360	11	18	0.846	2011
White MA	13	3.29	34/459	11	13	0.846	2011
Ivezaj V	11	3.20	5/127	7	11	0.700	2014
Masheb RM	11	3.14	63/453	10	11	0.526	2005
Hay P	10	2.28	62/238	7	10	0.350	2004
Lydecker JA	9	3.02	1/97	7	9	1.000	2017
Hoek HW	8	3.33	51/940	8	8	0.242	1991
Schmidt U	8	2.08	0/407	7	8	0.280	1999
Treasure J	8	1.63	9/541	7	8	0.250	1996

**Figure 5:**
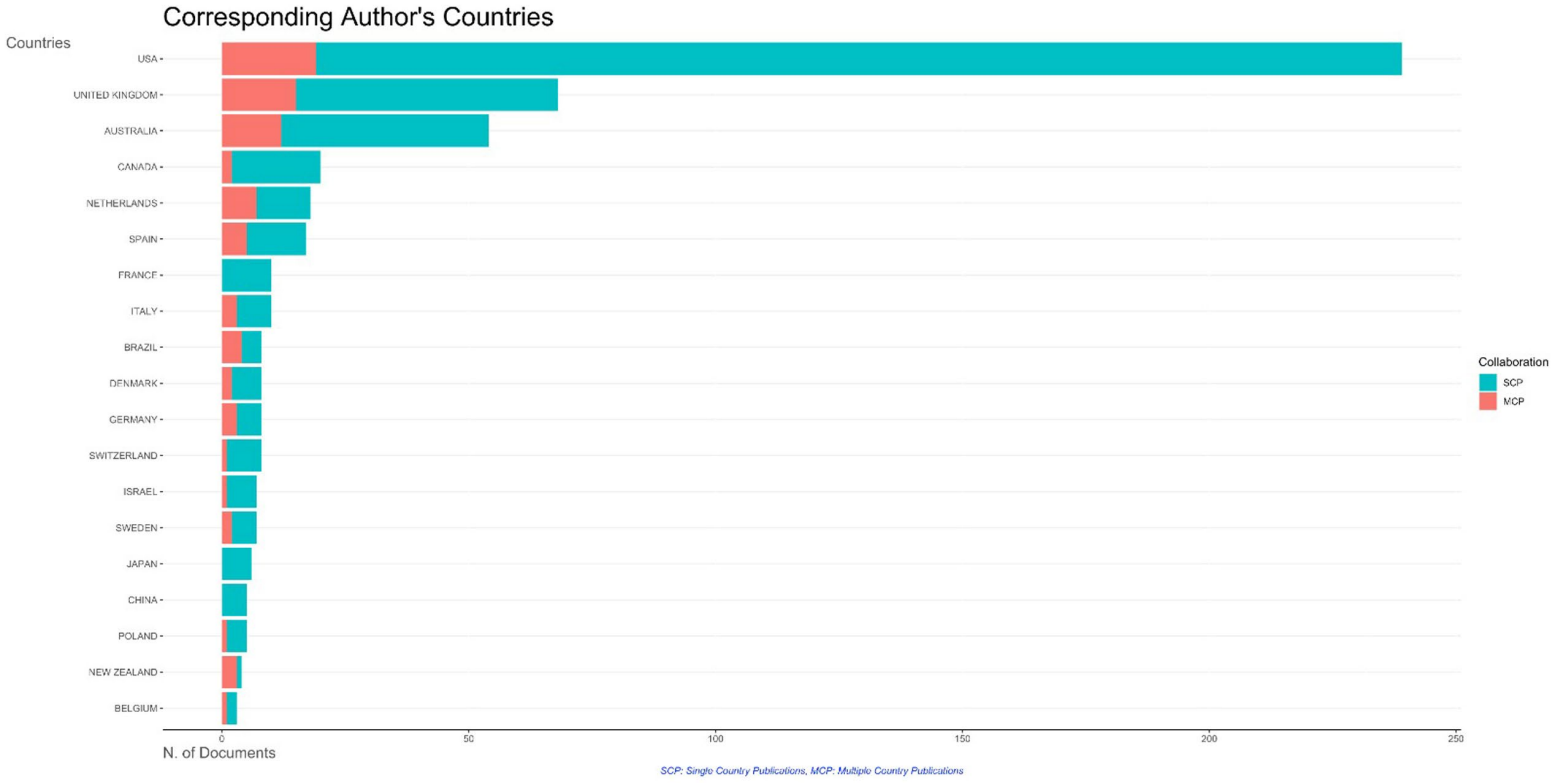
The most productive corresponding authors’ countries and their collaboration characteristics in the field.

**Figure 6:**
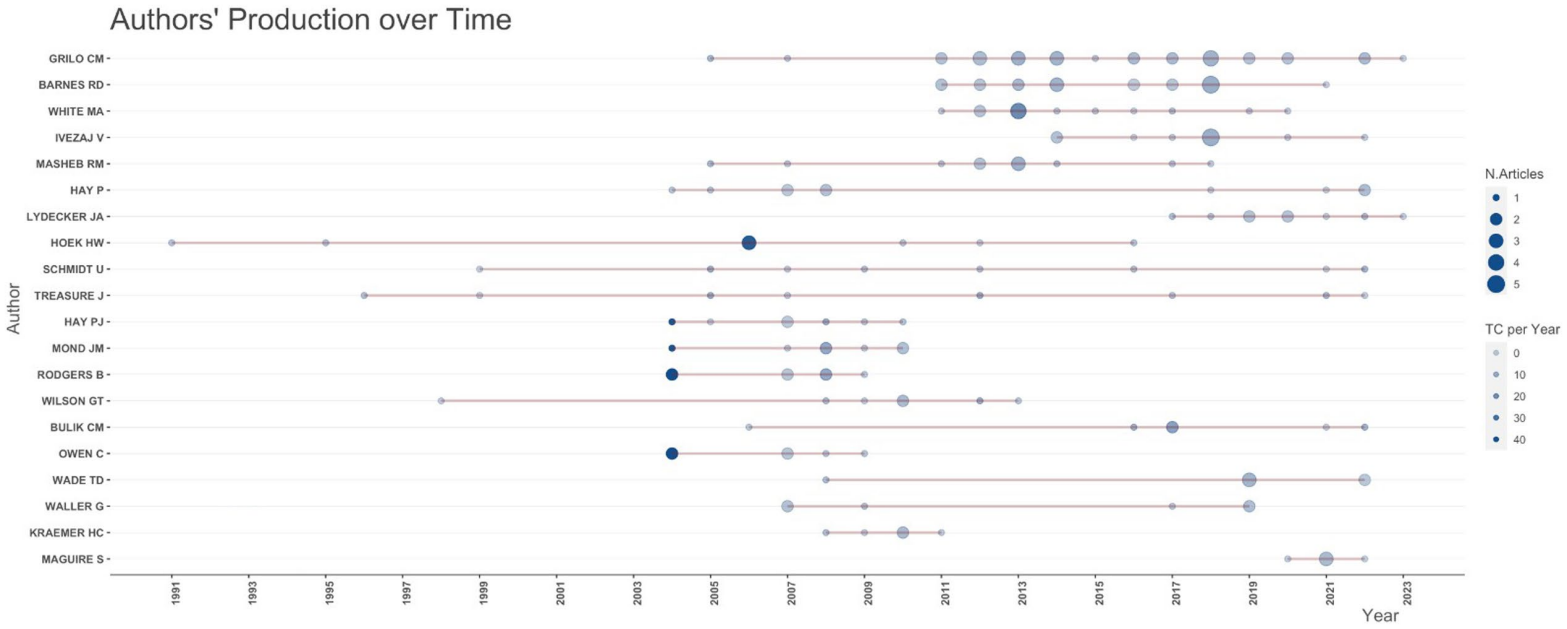
The authors’ production over time in the field.

Analysis of the most productive corresponding authors’ countries revealed that the United States produced the largest number of publications followed by the United Kingdom, Australia, and Canada. The scholars from France, Japan, and China are different in terms of collaboration as they only produced single-country publications (Figure 5). The top 10 authors’ productivities in the field of primary care-related EDs research over time are illustrated in Figure 6 with the numbers and corresponding years of publication.

### 3.3. Social structure and collaboration network analysis

A network analysis was performed to identify the essential authors regarding their centrality to the network (31). The author with the most prominent betweenness centrality was Grilo CM (Yale School of Medicine, United States), followed by Hay P (Western Sydney University, Australia), Barnes RD (Yale School of Medicine, United States), Rodgers B (Australian National University, Australia), and Mond JM (University of Tasmania, Australia). Eighteen clusters were identified by the network analysis. There were 11 authors in each of the first and second clusters. The top five authors in terms of betweenness centrality indices were part of these two clusters (Table 4). According to the closeness centrality, the five most central authors in the Scientometrics network were Bergh C (Karolinska Institute, Sweden), Brodin U (Mandometer Clinic, Sweden), Baxter J (University of Colorado, United States), Browne MAO (University of Otago, New Zealand), and Zimmerman M (Brown University, United States). Table 4 highlights the authors that ranked in the top 5 in at least one of the centrality measures (betweenness centrality, closeness centrality, and PageRank) including their relevant metrics and rankings in parenthesis.

**Table 4:** Indices of centrality for the top five authors in the network analysis of collaboration.

Authors^*^	Cluster	Betweenness centrality	Closeness centrality	PageRank
Grilo CM	1	17.72 (1)	0.08 (49)	0.04 (1)
Hay P	2	13.20 (2)	0.06 (57)	0.02 (8)
Barnes RD	1	12.20 (3)	0.08 (50)	0.03 (2)
Rodgers B	2	11.26 (4)	0.07 (52)	0.02 (4)
Mond JM	2	10.13 (5)	0.06 (55)	0.02 (5)
Bergh C	18	0.00 (32)	1.00 (1)	0.01 (30)
Brodin U	18	0.00 (33)	1.00 (2)	0.01 (31)
Baxter J	17	0.00 (34)	1.00 (3)	0.01 (32)
Browne MAO	17	0.00 (34)	1.00 (4)	0.01 (33)
Zimmerman M	16	0.00 (36)	1.00 (5)	0.01 (34)
Kraemer HC	5	6.94 (9)	0.14 (40)	0.02 (3)

^*^The authors are listed according to the betweenness centrality value.

### 3.4. Keywords analysis with bibliometrics

**Figure 7:**
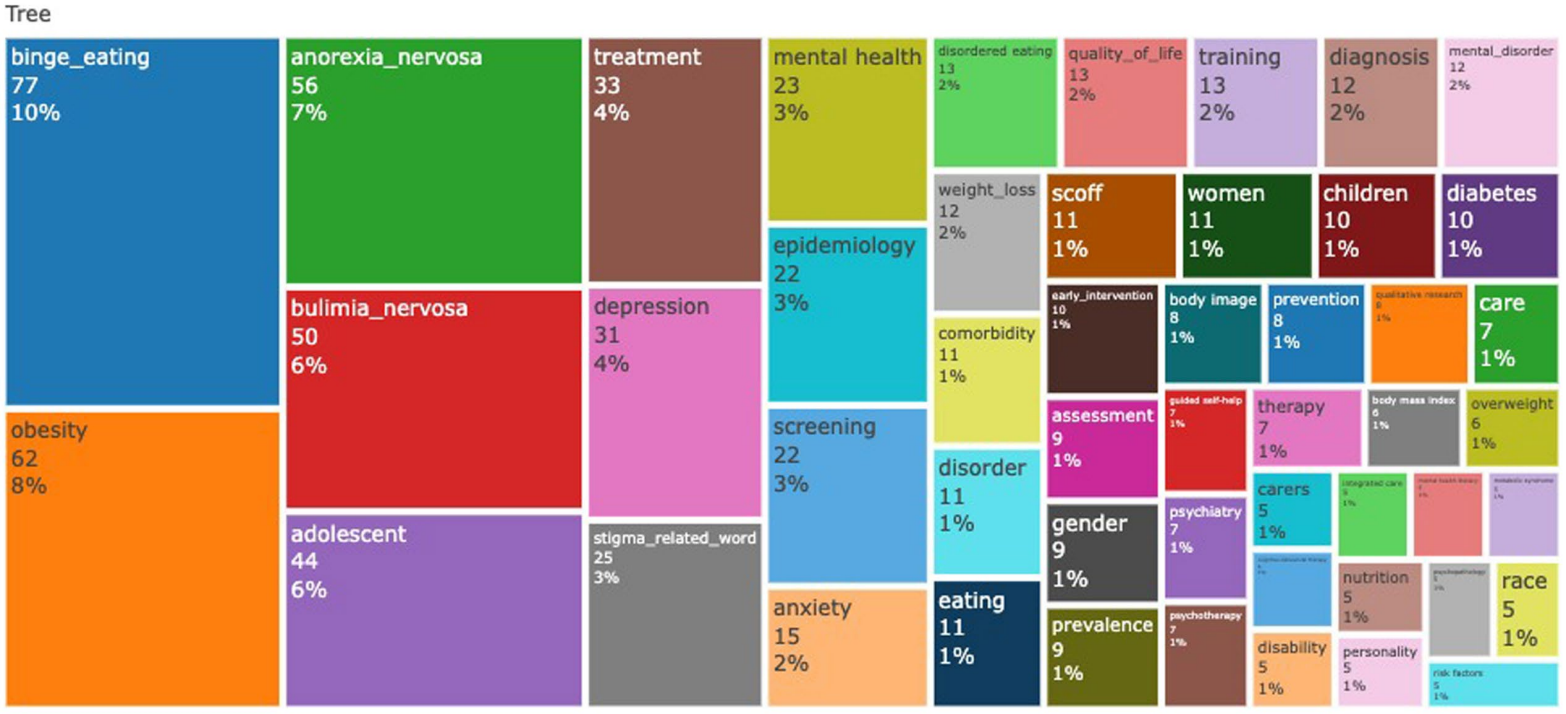
Tree map analysis of the authors’ keywords in the field.

The analysis of the most frequent authors’ keywords was calculated after removing the primary search queries (16% eating_disorder and 9% primary_care) and combining the stigma related words (meta-cognitive beliefs, inclusion beliefs, causal attribution, attitudes, low self-esteem, negative affect, ebullying, e-victimization, stereotypes, stereotypy, stigmatizing experiences, self-esteem, stigma, attitudes, weight bias, shame, and beliefs.) This was done to emphasize the visibility of scarce keywords that are all related to stigma. The words with more than 3% incidence were binge eating (10%), obesity (8%), anorexia nervosa (7%), bulimia nervosa (6%), adolescent (6%), treatment (4%), depression (4%), and stigma-related words (3%). The tree map of the keywords is given in the Figure 7.

### 3.5. Topic modeling analysis

The optimum number of groups used in the topic modeling analysis was determined to be 10 using the log-likelihood method. Of these groups, eight (screening, neurotic symptoms, training, adolescent, stigma, obesity-related conditions, family, and incidence) of them emerged as easily interpretable EDs related topics. It was not possible to group the two remaining groups with the least number of terms in a meaningful manner. The terms in these groups were patient, eating-disorder, symptoms, primary-care, depression, risk, diagnosis, incidence, treatment, pain, health, chronic, anorexia-nervosa, and medical. The identified topics are listed in Table 5 according to the number of appearances of the terms and presented with sample articles.

**Table 5:** Trend topics with representative publications according to the topic modeling.

Topics	Terms	Number of appearances	Sample articles
Screening	Eating-disorder, patient, scoff, questionnaire, primary-care, screening, positive, validity, eating, and clinical	1,603	Assessment and Treatment of Pediatric Eating Disorders: A Survey of Physicians and Psychologists. Robinson et al. (2012), Journal of The Canadian Academy of Child and Adolescent Psychiatry
			Four simple questions can help screen for eating disorders. Cotton, MA et al. (2003), Journal of General Internal Medicine
Neurotic symptoms	Disorder, anxiety, psychiatric, depression, patient primary care, women, eating disorder, prevalence, and mental disorder	1,177	Psychiatric disorders associated with the onset and persistence of bulimia nervosa and binge eating disorder during adolescence. Zaider TI et al. (2002), Journal of Youth and Adolescence
			The relationship between eating disorder psychopathology and health-related quality of life within a community sample. Vallance JK et al. (2011), Quality of Life Research
Training	Eating-disorder, training, primary-care, patient, health, treatment, screening, practice, adolescent, and medical	1,030	The effectiveness of a brief eating disorder training program in medical settings. Linville et al. (2013), Journal of Research in Nursing
			Turning eating disorders screening in primary practice into treatment: A clinical practice approach. Wade et al. (2022), International Journal of Eating Disorders
Adolescent	Eating-disorder, primary-care, adolescent, risk, patient, anorexia-nervosa, food, screening, disordered-eating, and health	857	Adolescents with a diagnosis of anorexia nervosa: Parents’ experience of recognition and deciding to seek help. Thomson et al. (2014), Clinical Child Psychology and Psychiatry
			Identifying Risk Factors for Disordered Eating among Female Youth in Primary Care. Russon et al. (2019), Child Psychiatry\Human Development
Stigma	Eating-disorder, weight, eating, stigma-related-word, obesity, problems, symptoms, primary-care, mental-health, and adolescent	503	An examination of weight bias among treatment-seeking obese patients with and without binge eating disorder. Barnes et al. (2014), General Hospital Psychiatry
			The role of stigma on the depressive symptoms of French women with overweight or obesity. Juhel et al. (2021), European Review of Applied Psychology
Obesity related conditions	Binge-eating, treatment, eating-disorder, patient, primary-care, obesity, weight-loss, self-help, eating, and outcomes	399	Self-esteem mediates the associations among negative affect, body disturbances, and interpersonal problems in treatment-seeking obese individuals. Salerno et al. (2015), Clinical Psychologist
			Primary care provider familiarity with binge eating disorder and implications for obesity management: A preliminary survey. Cummins et al. (2003), Journal of Clinical Psychology in Medical Settings
Family	Children, primary-care, physicians, family, parents, patient, acute, illness, age, and adolescent	235	Can adolescents with eating disorders be treated in primary care? A retrospective clinical cohort study. Lebow et al. (2021), Journal of Eating Disorders
			Effectiveness of delivering evidence-based eating disorder treatment via telemedicine for children, adolescents, and youth. Steinberg et al. (2022), Eating Disorders
Incidence	Patient, incidence, risk, eating-disorder, adults, primary-care, diagnosis, disease, bulimia-nervosa, and women	179	Incidence, prevalence, and mortality of anorexia nervosa and other eating disorders. Hoek et al. (2006), Current Opinion in Psychiatry
			Prevalence of mental and social disorders in adults attending primary care centers in Bosnia and Herzegovina. Broers et al. (2006) Croatian Medical Journal

**Figure 8:**
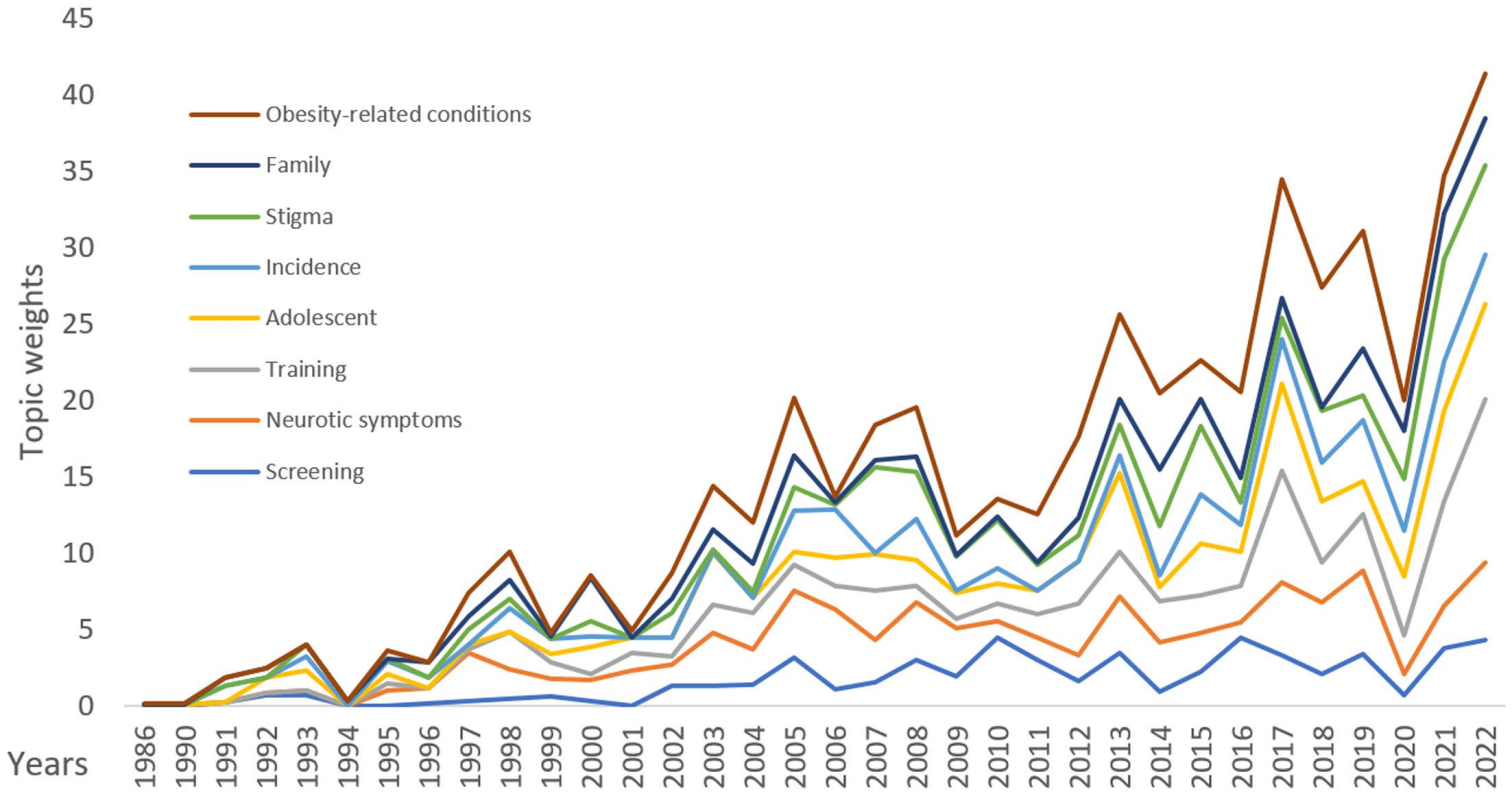
Distribution of the total weights of the topics by years.

Additionally, the visibility of the topics in the research area was analyzed over the years (Figure 8). After 2020, the trend generally increased in all topics. While screening and neurotic symptoms followed a relatively low trend; obesity-related conditions, family, and stigma topics’ trends were high.

## 4. Discussion

The main purpose of this study was to explore eating disorder studies from a primary care and stigma perspective. Therefore, the initial search was conducted with three class of queries related to EDs, primary care, and stigma. However, the number of documents obtained yielded a limited number of articles which was the first sign pointing to the gap in the field. Because this number was quite small for bibliometric analysis, the WoS search was focused only on primary care and eating disorder omitting stigma queries. Furthermore, a topic analysis was conducted in order to examine the obtained data in more depth and reveal a small number of stigma issues, and identify other research trends.

With the analysis of 541 journal articles published from 1986 to 2022, this bibliometric study aimed to provide a critical overview of how EDs research related to primary care has evolved over time and how the stigma-related concept has taken place in this field. Our results showed that the number of articles on EDs research related to primary care had increased steadily from 1986 to 2022. The upward tendency in publications indicates that the research in the field keeps growing, advancing, and attracting attention. Yet, the main finding of the study is that despite this linear increase over the years, the subject of stigma did not take a prominent place in the literature. Along with our findings, the United States Preventive Services Task Force and a systematic review also suggest that there is insufficient evidence to evaluate the benefits and harms of screening for eating disorders in primary care settings (32, 33). Further studies are needed to increase certainty about the potential benefits and harms, including overdiagnosis and stigma associated with screening results.

A recent bibliometric analysis of publications from the International Journal of Eating Disorders spanning the past 30 years revealed a significant surge in all studies conducted solely on eating disorders (14). Although our study was confined to primary care and included all the journals in the field, it is reasonable to assume that this spike in interest extends to all related areas.

During our analysis, we found that more than half of the articles were released in the final quarter of the assessed years. This indicates a growing interest in primary care-related EDs studies. However, the citation trend did not match the publication pattern. It peaked in 1995 and 2005 and dropped below the average citation value after 2015. In 2004, there was a significant increase in the number of publications, nearly three times the annual average, as Park et al. (14) discovered in their bibliometric study. The observed peak of citations in our study during the following year may be due to this increase production in the primary care-related EDs research area. Conducting further research on the reasons behind the decline in citation trends could help the field understand and improve their studies.

Our analysis found that one-third of all publications come from only 15 journals, which have made the most substantial scientific contributions to the field. The International Journal of Eating Disorders is a highly regarded publication for EDs. In May 2023, there were 27 journals in the “PHC” category on the WoS database. Since the PHC category is not specific to any particular organ systems or diseases/disorders, it is reasonable to assume that all 27 journals may be interested in EDs. However, only four of the top 25 journals are primary healthcare journals, with Psychiatry, Clinical Psychology, and General Internal Medicine being the most prominent publication areas, while Primary Health Care ranking seventh.

Comprehensive bibliometric analysis of all published research of He et al. (18) on EDs shows a consistent trend of increasing production in Western countries. These findings are supported by a recent bibliometric study investigating the top 100 most cited studies and network visualization of eating and feeding disorders research conducted by Shah et al. (17). Importance of global collaboration and the value of diverse perspectives in advancing knowledge and research is well-known. Yet, collaboration patterns among scholars from various countries show that there are potential opportunities for larger international and cross-cultural research.

An important metric for analyzing networks is “centrality,” which determines the most significant or central nodes within a network (31). Through network analysis, the crucial authors with high centrality to the rest of the network were identified. Closeness centrality is one of the centrality measures that identifies individuals who can reach others in the network quickly. This metric is based on the average shortest path length between a node and all other nodes in a network. Nodes with high closeness centrality are close to others in the network and can benefit from hearing their opinions more quickly. Based on closeness centrality, the five most central authors in the Scientometrics network were Bergh C (Karolinska Institute, Sweden), Brodin U (Mandometer Clinic, Sweden), Baxter J (University of Colorado, United States), Browne MAO (University of Otago, New Zealand), and Zimmerman M (Brown University, United States).

The second centrality measure is betweenness centrality. Nodes that exhibit high betweenness centrality can function as connectors, facilitating communication between isolated groups, while also acting as gatekeepers who regulate the flow of information. Additionally, research indicates that authors possessing high betweenness centrality tend to form more collaborations than researchers with high closeness centrality (25). Based on the measure of betweenness centrality, the author who stood out the most was Grilo CM (Yale School of Medicine, United States), followed by Hay P (Western Sydney University, Australia), Barnes RD (Yale School of Medicine, United States), Rodgers B (Australian National University, Australia), and Mond JM (University of Tasmania, Australia.)

To summarize, centrality measures can assist in recognizing experts in the primary care-related EDs field. This includes authors who have the ability to easily connect with other authors in the network (high in closeness), act as gatekeepers (high in-betweenness), or have connections with leading individuals (high in PageRank). The network analysis identified 11 clusters, with the first and second one being the largest. The top five authors in terms of betweenness centrality indices were part of these two clusters. Their collaboration results in this cluster were mainly focused on primary care and obesity, making it the most significant. These findings are also valuable in that they reinforce the first topic (obesity-related conditions) of the topic analysis.

The scope of this study is confined to academic literature available on databases such as the WoS. Consequently, sources from the gray literature and lay journals have been omitted due to their likelihood of containing unofficial or unpublished material that is not deemed suitable for academic research. It is important to note that the study’s findings may not encompass any relevant information that can be found in the gray literature. Furthermore, this study acknowledges the limitations associated with the use of WoS or similar databases. Specifically, these databases may have limitations in terms of coverage, particularly in certain subject areas or language groups. These limitations could lead to the oversight of some significant studies and result in findings that are based on a limited perspective. However, these databases remain valuable resources for providing a general overview and establishing a comprehensive foundation. To address such limitations, it is essential to incorporate different sources and methods to contextualize our findings within a broader framework.

The issue of stigma in eating disorders is a significant and complex problem. Primary care professionals possess a crucial role in addressing this matter through early intervention, as they are strategically positioned for early diagnosis and preventive medicine practices. Nonetheless, research in this field is limited, as evidenced by the results of this study. Additionally, it is imperative to acknowledge the significant contributions of the relevant scientific societies. These organizations have played a pivotal role in advancing our understanding of eating disorders and stigma by fostering collaboration, disseminating research findings, and advocating for evidence-based practices. Their efforts have been instrumental in bridging the gap between research and practice, ensuring that the latest research in the field reaches clinicians, policymakers, and the broader public. Apart from the research gap identified by this bibliometric analysis, the cooperation network and pioneering research groups can serve as valuable resources for young researchers and new projects.

In conclusion, it is crucial to pay more attention to identifying individuals with EDs at an early stage, providing them with appropriate treatment, and addressing the societal stigma associated with this condition. Primary care can play a vital role in detecting EDs in its early stages, but there is a lack of research in community-based settings. Further exploration is necessary to investigate routine EDs screening in primary care, while considering the impact of stigma. It is essential to note that false positive results from EDs screening may contribute to external or internal stigma. Therefore, primary care providers need specialized training and resources to manage EDs patients effectively.

The lack of research on the stigma associated with EDs in primary care is a concerning issue that requires attention. Although some efforts have been made to overcome this problem recently, it is evident that further studies are necessary to gain a comprehensive understanding of this complex issue. By conducting more research, effective strategies can be developed to combat the stigma and provide better care for individuals struggling with EDs. The leading research groups as identified in our study may play a crucial role in the expansion of primary care related EDs research in terms of ending the mental health stigma.

## Data availability statement

The original contributions presented in the study are included in the article/**Supplementary material**, further inquiries can be directed to the corresponding author.

## Author contributions

HK and SÖ: conception and design of the study. HK, ET, ZÖ, and AN: investigation and database organization. ET and HK: statistical analysis, software, and writing the first draft of the manuscript. All authors contributed to the article and approved the submitted version.

## References

[1] American Psychiatric Association. DSM-5. Washington, DC: American Psychiatric Association (2013).

[2] Castelpietra G Knudsen AK Agardh EE Armocida B Beghi M Iburg KM. The burden of mental disorders, substance use disorders and self-harm among young people in Europe, 1990–2019: findings from the global burden of disease study 2019. Lancet Reg Health Eur. (2022) 16:100341. doi: 10.1016/j.lanepe.2022.100341, PMID: 35392452 PMC8980870

[3] BEAT Delaying for years, denied for months. (2017). Available at: https://www.beateatingdisorders.org.uk/uploads/documents/2017/11/delaying-for-years-denied-for-months.pdf (Accessed June 20, 2023).

[4] Keski-Rahkonen A Mustelin L. Epidemiology of eating disorders in Europe: prevalence, incidence, comorbidity, course, consequences, and risk factors. Curr Opin Psychiatry. (2016) 29:340–5. doi: 10.1097/YCO.0000000000000278, PMID: 27662598

[5] Klein DA Sylvester J Schvey NA. Eating disorders in primary care: diagnosis and management. Am Fam Physician. (2021) 103:22–32. PMID: 33382560

[6] Allen J Gay B Crebolder H Heyrman J Svab I Ram P. The European definitions of the key features of the discipline of general practice: the role of the GP and core competencies. Br J Gen Pract. (2002) 52:526. PMID: 12051237 PMC1314348

[7] Ebneter DS Latner JD. Stigmatizing attitudes differ across mental health disorders: a comparison of stigma across eating disorders, obesity, and major depressive disorder. J Nerv Ment Dis. (2013) 201:281–5. doi: 10.1097/NMD.0b013e318288e23f, PMID: 23538972

[8] Sangvai D. Eating disorders in the primary care setting. Prim Care. (2016) 43:301–12. doi: 10.1016/j.pop.2016.01.007, PMID: 27262009

[9] Mensinger JL Calogero RM Stranges S Tylka TL. A weight-neutral versus weight-loss approach for health promotion in women with high BMI: a randomized-controlled trial. Appetite. (2016) 105:364–74. doi: 10.1016/j.appet.2016.06.006, PMID: 27289009

[10] Brelet L Flaudias V Désert M Guillaume S Llorca PM Boirie Y. Stigmatization toward people with anorexia nervosa, bulimia nervosa, and binge eating disorder: a scoping review. Nutrients. (2021) 13:2834. doi: 10.3390/nu13082834, PMID: 34444994 PMC8400545

[11] Börner K Chen C Boyack KW. Visualizing knowledge domains. Annu Rev Inf Sci Technol. (2003) 37:179–255. doi: 10.1002/aris.1440370106

[12] Soh NLW Waller G. Publications on cross-cultural aspects of eating disorders. J Eat Disord. (2013) 1:1–4. doi: 10.1186/2050-2974-1-4 24764527 PMC3776204

[13] Strand M Bulik CM. Trends in female authorship in research papers on eating disorders: a 20-year bibliometric study. BJPsych Open. (2018) 4:39–46. doi: 10.1192/bjo.2017.8, PMID: 29467058 PMC6020273

[14] Park E Kim WH. A retrospective literature review of eating disorder research (1990–2021): application of bibliometrics and topical trends. Int J Environ Res Public Health. (2022) 19:7710. doi: 10.3390/ijerph19137710, PMID: 35805366 PMC9265657

[15] Almenara CA. 40 years of research on eating disorders in domain-specific journals: Bibliometrics, network analysis, and topic modeling. PLoS One. (2022) 17:e0278981. doi: 10.1371/journal.pone.0278981, PMID: 36520823 PMC9754234

[16] Sa'ed HZ Shakhshir M Abushanab AS Koni A Shahwan M Jairoun AA. Mapping the landscape and structure of global research on binge eating disorder: visualization and bibliometric analysis. World J Psychiatry. (2022) 12:982. doi: 10.5498/wjp.v12.i7.982, PMID: 36051594 PMC9331445

[17] Shah MW Ahmad T Khan M Sun G. Global research trends, top-100 most cited studies, and network visualization of eating and feeding disorders research from 1900-2020: a historical bibliometric analysis. Electron J Gen Med. (2022) 19:em368. doi: 10.29333/ejgm/11839

[18] He J Kang J Sun S Cooper M Zickgraf HF Zhai Y. The landscape of eating disorders research: a 40-year bibliometric analysis. Eur Eat Disord Rev. (2022) 30:96–109. doi: 10.1002/erv.2884, PMID: 35040236

[19] Grover S Gupta BM. Eating disorders research in India: a bibliometric assessment of publications output during 2000–2019. J Ment Health Hum Behav. (2021) 26:74. doi: 10.4103/jmhhb.jmhhb_145_20

[20] Moed HF. Citation Analysis in Research Evaluation. Netherlands: Springer Science & Business Media (2005).

[21] R Core Team R: A language and environment for statistical computing. R foundation for statistical computing, Vienna, Austria (2023). Available at: https://www.R-project.org/ (Accessed May 20, 2023).

[22] Aria M Cuccurullo C. Bibliometrix: an R-tool for comprehensive science mapping analysis. J Inf Secur. (2017) 11:959–75. doi: 10.1016/j.joi.2017.08.007, PMID: 37427124

[23] Blondel VD Guillaume JL Lambiotte R Lefebvre E. Fast unfolding of communities in large networks. J Statist Mech Theory Exper. (2008) 10:P10008. doi: 10.1088/1742-5468/2008/10/P10008

[24] Brin S Page L. The anatomy of a large-scale hypertextual web search engine. Comput Netw ISDN Syst. (1998) 30:107–17.

[25] Milojević S. Network analysis and indicators In: Measuring Scholarly Impact: Methods and Practice Ding Y Rousseau R Wolfram D, eds. (Switzerland: Springer International Publishing) (2014). 57–82.

[26] Wickham H François R Henry L Müller K. dplyr: a grammar of data manipulation. R package version 1.0.7. (2021). Available at: https://CRAN.R-project.org/package=dplyr (Accessed June 20, 2023).

[27] Wickham H. stringr: Simple, consistent wrappers for common string operations. R package version 1.4.1. (2019). Available at: https://CRAN.R-project.org/package=stringr (Accessed June 20, 2023).

[28] Chang J. lda: Collapsed Gibbs sampling methods for topic models. R package version 1.4.3. (2019). Available at: https://CRAN.R-project.org/package=lda (Accessed June 20, 2023).

[29] Blei DM Ng AY Jordan MI. Latent Dirichlet allocation. J Mach Learn Res. (2003) 3:993–1022.

[30] Griffiths TL Steyvers M. Finding scientific topics. Proc Natl Acad Sci. (2004) 101:5228–35. doi: 10.1073/pnas.030775210 14872004 PMC387300

[31] Freeman LC. Centrality in social networks conceptual clarification. Soc Networks. (1979) 1:215–39. doi: 10.1016/0378-8733(78)90021-7

[32] US Preventive Services Task Force. Screening for eating disorders in adolescents and adults: US preventive services task force recommendation statement. J Am Med Assoc. (2022) 327:1061–7. 10.1001/jama.2022.1806 35289876

[33] Peat CM Feltner C. Addressing eating disorders in primary care: understanding screening recommendations and opportunities to improve care. Int J Eat Disord. (2022) 55:1202–7. doi: 10.1002/eat.23786, PMID: 35903970

