# Supplementary material for: Eating disorders, primary care, and stigma: an analysis of research trends and patterns
Source: Front Psychiatry. 2023 Sep 29;14:1243922. doi: 10.3389/fpsyt.2023.1243922 (PMC10570408; doi:10.3389/fpsyt.2023.1243922)
Supplement: Supplementary file 1 [file Data_Sheet_1.PDF]

````
---
title: 'Eating Disorders, Primary Care, and Stigma: An Analysis of
 Research Trends
  and Patterns'
author: "Erkan TİYEKLİ"
date: "2023-07-24"
output: html_document
---

```{r setup, include=FALSE}
knitr::opts_chunk$set(echo = TRUE)
```

## R Markdown

This is an R Markdown document. Markdown is a simple formatting syntax
 for authoring HTML, PDF, and MS Word documents. For more details on
 using R Markdown see <http://rmarkdown.rstudio.com>.

When you click the **Knit** button a document will be generated that
 includes both content as well as the output of any embedded R code
 chunks within the document. You can embed an R code chunk like this:

```{r cars}
summary(cars)
```

## Including Plots

You can also embed plots, for example:

```{r pressure, echo=FALSE}
plot(pressure)
```

Note that the `echo = FALSE` parameter was added to the code chunk to
 prevent printing of the R code that generated the plot.

# Load Libraries
library(topicmodels)
library(tm)
library(bibliometrix)
library(text2vec)
library(RefManageR)
library(ggplot2)

# Read Bibliographic Data
file_path <- "e:/Bibfile_541.bib"
````

```r
bib_data <- ReadBib(dosya_yolu)


filter_terms <- c("eating disorder", "stigma")

# Create the dataset
corpus <- Corpus(VectorSource(paste(bib_data$title, bib_data$keywords,
 bib_data$abstract, sep = " ")))

# Data Preprocessing
corpus <- tm_map(corpus, content_transformer(tolower))
corpus <- tm_map(corpus, removePunctuation)
corpus <- tm_map(corpus, removeNumbers)
corpus <- tm_map(corpus, removeWords, stopwords("english"))
corpus <- tm_map(corpus, stripWhitespace)

# Word Replacement
# Several term replacements are performed to standardize certain terms.
# Example: "eating disorder" is replaced with "eating_disorder."
corpus <- tm_map(corpus, content_transformer(gsub), pattern = "eating
 disorder", replacement = "eating_disorder")

# (Other similar term replacements are done here)

corpus <- tm_map(corpus, content_transformer(gsub), pattern = "eating
 disorders", replacement = "eating_disorder")

corpus <- tm_map(corpus, content_transformer(gsub), pattern = "body
 image", replacement = "body_image")

corpus <- tm_map(corpus, content_transformer(gsub), pattern = "body mass
 index", replacement = "body_mass_index")

corpus <- tm_map(corpus, content_transformer(gsub), pattern =
 "borderline personality disorder", replacement =
 "borderline_personality")

corpus <- tm_map(corpus, content_transformer(gsub), pattern =
 "cognitive-behavioral therapy", replacement =
 "cognitive_behavioral_therapy")

corpus <- tm_map(corpus, content_transformer(gsub), pattern = "early
 intervention", replacement = "early_intervention")

corpus <- tm_map(corpus, content_transformer(gsub), pattern = "emergency
 department", replacement = "emergency_department")

corpus <- tm_map(corpus, content_transformer(gsub), pattern = "general
```

```
practice", replacement = "general_practice")

corpus <- tm_map(corpus, content_transformer(gsub), pattern = "guided
self-help", replacement = "guided_self_help")

corpus <- tm_map(corpus, content_transformer(gsub), pattern =
"guided_self_help", replacement = "guided_self_help")

corpus <- tm_map(corpus, content_transformer(gsub), pattern = "health
services", replacement = "health_services")

corpus <- tm_map(corpus, content_transformer(gsub), pattern =
"integrated care", replacement = "integrated_care")

corpus <- tm_map(corpus, content_transformer(gsub), pattern = "irritable
bowel syndrome", replacement = "irritable_bowel_syndrome")

corpus <- tm_map(corpus, content_transformer(gsub), pattern = "mental
health literacy", replacement = "mental_health_literacy")

corpus <- tm_map(corpus, content_transformer(gsub), pattern = "mental
health", replacement = "mental_health")

corpus <- tm_map(corpus, content_transformer(gsub), pattern =
"qualitative research", replacement = "qualitative_research")

corpus <- tm_map(corpus, content_transformer(gsub), pattern = "risk
factors", replacement = "risk_factors")

corpus <- tm_map(corpus, content_transformer(gsub), pattern = "self-
esteem", replacement = "self_esteem")

corpus <- tm_map(corpus, content_transformer(gsub), pattern = "self-
help", replacement = "self_help")

corpus <- tm_map(corpus, content_transformer(gsub), pattern = "substance
use", replacement = "substance_use")

corpus <- tm_map(corpus, content_transformer(gsub), pattern = "weight
loss", replacement = "weight_loss")

corpus <- tm_map(corpus, content_transformer(gsub), pattern = "weight
management", replacement = "weight_management")

corpus <- tm_map(corpus, content_transformer(gsub), pattern =
"patients", replacement = "patient")
```

```
# Merge multiple words into a single word
corpus <- tm_map(corpus, content_transformer(function(x) gsub("\
 \b(adolescence|adolescent|adolescents|children and adolescents)\\b",
 "adolescent", x)))

# This line of code utilizes regular expressions to find variations of
 the specified terms (adolescence, adolescent, adolescents, children and
 adolescents)
and replaces them with the word "adolescent" using the gsub function.
 This transformation helps in standardizing the different forms of the
 same concept,
making it easier to analyze and model the data.

corpus <- tm_map(corpus, content_transformer(function(x) gsub("\
 \b(anorexia nervosa|anorexia)\\b", "anorexia_nervosa", x)))

corpus <- tm_map(corpus, content_transformer(function(x) gsub("\
 \b(anxiety|anxiety disorders)\\b", "anxiety", x)))

corpus <- tm_map(corpus, content_transformer(function(x) gsub("\\b(bed|
 bingeeating_disorder|binge_eating|binge|binge eating|binge eating
 disorder|binge-eating disorder)\\b", "binge_eating", x)))

corpus <- tm_map(corpus, content_transformer(function(x) gsub("\
 \b(bulimia|bulimia nervosa)\\b", "bulimia_nervosa", x)))

corpus <- tm_map(corpus, content_transformer(function(x) gsub("\
 \b(disorders|disorder)\\b", "disorder", x)))

corpus <- tm_map(corpus, content_transformer(function(x) gsub("\
 \b(diabetes|diabetes mellitus|type 2 diabetes)\\b", "diabetes", x)))

corpus <- tm_map(corpus, content_transformer(function(x) gsub("\
 \b(child|children)\\b", "children", x)))

corpus <- tm_map(corpus, content_transformer(function(x) gsub("\
 \b(disordered eating)\\b", "disordered_eating", x)))

corpus <- tm_map(corpus, content_transformer(function(x) gsub("\
 \b(eating_disorder|eating_disorders)\\b", "eating_disorder", x)))

corpus <- tm_map(corpus, content_transformer(function(x) gsub("\\b(early
 diagnosis|early intervention)\\b", "early_intervention", x)))

corpus <- tm_map(corpus, content_transformer(function(x) gsub("\
 \b(general practice|general practitioners|general practitioner|primary
 care|primary|primary health care|primary care providers|general
 practitioner)\\b", "primary_care", x)))
```

```
corpus <- tm_map(corpus, content_transformer(function(x) gsub("\
 \b(health-related quality of life|quality of life)\\b",
 "quality_of_life", x)))

corpus <- tm_map(corpus, content_transformer(function(x) gsub("\
 \b(mental disorder|mental disorders)\\b", "mental_disorder", x)))

corpus <- tm_map(corpus, content_transformer(function(x) gsub("\
 \b(primary|primary care|primary health care)\\b", "primary_care", x)))

corpus <- tm_map(corpus, content_transformer(function(x) gsub("\
 \b(validation|validity)\\b", "validity", x)))


# Replace stigma-related words with "stigma_related_word"
stigma_words <- c("prejudices", "weightbias", "bias", "biases",
 "stereotype", "stereotypes", "stereotypical", "stereotypy", "victim",
 "victimization", "victims", "blame", "shame", "bullied", "bullying",
 "belief", "beliefs", "believe", "believed", "behaviorsbeliefs",
 "assuming", "assumption", "assumptions", "attributable", "attributed",
 "attribution", "attributions", "attitude", "attitudes", "perception",
 "perceptions", "selfesteem", "selfimage", "esteem", "selfacceptance",
 "selfacceptance", "selfimage", "attitude", "attitudes", "attitudinal",
 "meta-cognitive beliefs", "inclusion beliefs", "causal attribution",
 "low self-esteem", "negative affect", "ebullying", "e-victimization",
 "stigmatizing experiences", "stigma", "weight bias", "shame", "beliefs")

corpus <- tm_map(corpus, content_transformer(function(x)
 gsub(paste(stigma_words, collapse = "|"), "stigma_related_word", x)))

# Remove specific words from the text corpus
corpus <- tm_map(corpus, content_transformer(function(x) gsub("\
 \bresults\\b|\\byears\\b|\\bparticipants\\b|\\bcase\\b|\\bstudy\\b|\
 \bnull\\b|\\bsignificantly\\b|\\beds\\b|\\bgps\\b|\\bcan\\b|\\bamong\\b|
 \\buse\\b|\\bcare\\b|\\bmay\\b|\\bassociated\\b|\\bveterans\\b|\
 \bveteran\\b", "", x)))

# Remove specific words "primary_care" and "eating_disorder" from the
 text corpus
corpus <- tm_map(corpus, content_transformer(function(x) gsub("\
 \bprimary_care\\b|\\beating_disorder\\b", "", x)))


# Create Document-Term Matrix (DTM)
dtm <- DocumentTermMatrix(corpus)
#In this code, the function DocumentTermMatrix() is used to convert the
 text corpus (corpus) into a Document-Term Matrix (DTM). The DTM is a
```

mathematical representation of the text corpus, where each row represents a document (in this case, each entry in the corpus is considered a separate document), and each column represents a unique term (word) in the corpus. The cells of the matrix contain the term frequency (number of times a term appears) in each document.
After executing this code, the variable dtm will store the Document-Term Matrix, which can be used for various text analysis and natural language processing tasks.

```
# Evaluate log-likelihood for different values of "k" (number of topics)
 to determine the optimum topic number
k_values <- c(2:10)
log_likelihood <- rep(0, length(k_values))

for (i in 1:length(k_values)) {
  k <- k_values[i]
  lda_model <- LDA(dtm, k = k)
  log_likelihood[i] <- logLik(lda_model)
}
```

#In this code, a vector k_values is created containing the values from 2 to 10, which represent the potential number of topics to be considered for topic modeling. The log_likelihood vector is initialized to zeros and will store the log-likelihood values for each corresponding k value.
The objective of this code is to loop over each value of k, create an LDA (Latent Dirichlet Allocation) model with that specific number of topics, calculate its log-likelihood, and store it in the log_likelihood vector. The log-likelihood is a measure of how well the LDA model fits the data. The higher the log-likelihood, the better the model is at explaining the observed data.
After running this code, the log_likelihood vector will contain log-likelihood values for each k, which can be used to determine the optimum number of topics (i.e., the k value with the highest log-likelihood) for the LDA model.

```
# Determine the optimum number of topics (optimal_k) based on the
 highest log-likelihood value
optimal_k <- k_values[which.max(log_likelihood)]
```

#In this code, which.max(log_likelihood) is used to find the index of the highest log-likelihood value in the log_likelihood vector. The function which.max() returns the position of the maximum value in a vector. Then, k_values[...] is used to retrieve the corresponding k value from the k_values vector, representing the number of topics that resulted in the highest log-likelihood.

After running this code, the variable optimal_k will store the value of k that corresponds to the optimum number of topics for the LDA model, as determined by the highest log-likelihood value. This optimal_k value will be used to build the final LDA model with the best number of topics for the given text corpus

```
# Create the LDA model with the optimum number of topics (optimal_k)
lda_model <- LDA(dtm, k = optimal_k)
```

#In this code, the LDA() function from the topicmodels package is used to build the LDA model. The function takes two main arguments:
dtm: The Document-Term Matrix representing the text corpus.
k: The number of topics (optimal_k) that was determined as the best fit based on the highest log-likelihood value.
The LDA() function creates the LDA model with the specified number of topics using the given document-term matrix. This model will be used to discover the latent topics in the corpus and their associated word distributions.
After executing this code, the variable lda_model will store the LDA model, which can be further analyzed to explore the topics and their corresponding word distributions in the given text corpus.

```
# Display the terms representing the topics in the LDA model
terms <- terms(lda_model)


# Display the topics and their top 10 most frequent terms in the LDA model
topics <- terms(lda_model, 10)

# Print and display the topics along with their top 10 most frequent terms in the LDA model
for (i in 1:10) {
  cat("Topic", i, ": ", paste(topics[[i]], collapse = ", "), "\n")
}



# Convert topic weights into a data frame
topic_data <- data.frame(
  Topic = rep(paste("Topic", 1:optimal_k), each = nrow(topic_weights)),
  Weight = as.vector(topic_weights)
)
```

```
# Create the bar plot for topic weights
ggplot(topic_data, aes(x = Topic, y = Weight, fill = Topic)) +
  geom_bar(stat = "identity") +
  labs(title = "Topic Weights", x = "Topic", y = "Weight") +
  theme_minimal() +
  theme(legend.position = "none")


# Print and display the terms and their weights for each topic in the
 LDA model
topics <- terms(lda_model, 10)
topic_weights <- as.data.frame(lda_model@gamma)

for (i in 1:optimal_k) {
  cat("Topic", i, ":\n")
  for (j in 1:10) {
    term <- topics[[i]][j]
    weight <- topic_weights[j, i]
    cat(term, ": ", weight, "\n")
  }
  cat("\n")
}


# Finding the weight of the "Eating Disorder" topic
eating_disorder_terms <- topic_terms[[1]]
eating_disorder_weights <- topic_weights[, 1]  # Select the first column

# Printing terms and weights
for (i in 1:length(eating_disorder_terms)) {
  cat(eating_disorder_terms[i], ": ", eating_disorder_weights[i], "\n")
}



# Find the number of documents where the term "eating_disorder" appears
eating_disorder_freq <- sum(dtm[, "eating_disorder"])
cat("eating_disorder terimi", eating_disorder_freq, "kez geçmiştir.\n")

# Find the number of documents where the term "stigma_related_word"
 appears
stigma_related_word_freq <- sum(dtm[, "stigma_related_word"])
cat("stigma_related_word terimi", stigma_related_word_freq, "kez
 geçmiştir.\n")


# Create a matrix containing the terms and their weights
```

```r
topic_terms <- terms(lda_model, 10)

# Calculate the total word counts
total_counts <- colSums(as.matrix(dtm))

# Calculate the total word counts in each topic
topic_totals <- sapply(topic_terms, function(x) sum(total_counts[x]))

# Print the total word counts in each topic
for (i in 1:optimal_k) {
  cat("Topic", i, "kelime toplamı:", topic_totals[i], "\n")
}


# Convert topic distributions with years into a data frame
df <- data.frame(years = as.character(years), topic_distributions)

# Calculate the topic distribution by year
topic_distribution_by_year <- aggregate(. ~ years, data = df, FUN = sum)

# Print the topic distribution by year
print(topic_distribution_by_year)

# Visualize the topic distribution by year with a bar plot
barplot(as.matrix(topic_distribution_by_year[-1]), beside = TRUE,
        legend = TRUE, xlab = "Years", ylab = "Topic Weight")
```
