# Supplementary material for: Eating disorders, primary care, and stigma: an analysis of research trends and patterns
Source: Front Psychiatry. 2023 Sep 29;14:1243922. doi: 10.3389/fpsyt.2023.1243922 (PMC10570408; doi:10.3389/fpsyt.2023.1243922)
Supplement: Supplementary file 2 [file Data_Sheet_2.PDF]

````
---
title: 'Eating Disorders, Primary Care, and Stigma: An Analysis of
 Research Trends
  and Patterns'
author: "Erkan TİYEKLİ"
date: "2023-07-24"
output: html_document
---

```{r setup, include=FALSE}
knitr::opts_chunk$set(echo = TRUE)
```

## R Markdown

This is an R Markdown document. Markdown is a simple formatting syntax
 for authoring HTML, PDF, and MS Word documents. For more details on
 using R Markdown see <http://rmarkdown.rstudio.com>.

When you click the **Knit** button a document will be generated that
 includes both content as well as the output of any embedded R code
 chunks within the document. You can embed an R code chunk like this:

```{r cars}
summary(cars)
```

## Including Plots

You can also embed plots, for example:

```{r pressure, echo=FALSE}
plot(pressure)
```

Note that the `echo = FALSE` parameter was added to the code chunk to
 prevent printing of the R code that generated the plot.

# Load Libraries
library(bibliometrix)

#Please note that our software is open source and available for use,
 distributed under the MIT license.
When it is used in a publication, we ask that authors properly cite the
 following reference:
Aria, M. & Cuccurullo, C. (2017) bibliometrix: An R-tool for
 comprehensive science mapping analysis,
                       Journal of Informetrics, 11(4), pp 959-975,
````

```
 Elsevier.
Failure to properly cite the software is considered a violation of the
 license.
For information and bug reports:
                        - Take a look at https://www.bibliometrix.org
                        - Send an email to info@bibliometrix.org
                        - Write a post on https://github.com/
 massimoaria/bibliometrix/issues
Help us to keep Bibliometrix and Biblioshiny free to download and use by
 contributing with a small donation to support our research team
 (https://bibliometrix.org/donate.html)
To start with the Biblioshiny app, please digit:
#biblioshiny()


biblioshiny()
Installing mandatory package: shiny

Listening on http://127.0.0.1:4823
```
